# Rhinosinusitis in hematopoietic stem cell-transplanted patients: influence of nasosinus mucosal abnormalities?

**DOI:** 10.1186/scrt523

**Published:** 2014-12-04

**Authors:** Erica Ortiz, Albina Altemani, Afonso Celso Vigorito, Eulalia Sakano, Ester Maria Danielli Nicola

**Affiliations:** Otolaryngology Head Neck Department, University of Campinas, Campinas, Sao Paulo Brazil; Pathology Department, University of Campinas, Campinas, Sao Paulo Brazil; Hematology Department, University of Campinas, Campinas, Sao Paulo Brazil; Faculdade de Ciencias Médicas, Caixa Postal 6111 – Cidade Universitária ‘Zeferino Vaz’, Campinas, São Paulo Brazil

## Abstract

**Introduction:**

Rhinosinusitis is characterized by inflammation extending from the mucosa of the nasal cavity into the paranasal sinuses. There are some aggravating features, such as immunosuppression, that can cause the nasal mucosal inflammation to linger for a long period, resulting in chronic or recurrent episodes. Such immunosuppression is the major feature of patients undergoing a hematopoietic stem cell transplant (HSCT); rhinosinusitis prevalence is higher in this group compared to immunocompetent patients. Nasal epithelial abnormalities have been described in, and may have some influence over, recurrent sinus infections among those patients. However, it is not clear whether rhinosinusitis can trigger mucosal abnormalities or whether a preexisting vulnerability for sinusitis recurrence is more likely. The objective of the study was to verify the influence of rhinosinusitis on nasal epithelial damage in patients undergoing hematopoietic stem cell transplantation.

**Method:**

A total of 30 allogeneic HSCT patients were divided into two groups: 24 patients with chronic or recurrent rhinosinusitis and 6 patients without rhinosinusitis. These patients underwent a biopsy of the uncinate process that was analyzed by transmission electron microscopy and optical microscopy.

**Results:**

The nasal mucosa analysis by optical microscopy showed no significant abnormalities. The ciliary orientation was obviously normal in the transplanted patients without rhinosinusitis. There was a trend toward a difference in the amount of cilia (decreased) and the primary modification of the ultrastructure of transplanted patients with rhinosinusitis.

**Conclusion:**

HSCT patients, with and without rhinosinusitis, showed no significant histological abnormalities, except for ciliary disorientation and a possible decrease in ciliary and ultrastructural abnormalities in HSCT patients with rhinosinusitis.

## Introduction

Rhinosinusitis is characterized by inflammation extending from the mucosa of the nasal cavity into the paranasal sinuses, blocking the sites of sinus drainage, especially the middle meatus [1]. The uncinate process is a crucial anatomical structure of the middle meatus, and its mucosa may be representative of the paranasal sinuses [2, 3]. Such a blockage in the uncinate process region can trigger secretion stasis within the maxillary, frontal and ethmoid sinuses, thus altering mucociliary clearance to favor the possibility of microorganism proliferation and nasal mucosa infections [1, 3].

Certain aggravating factors can extend nasal mucosa inflammation for longer periods, constituting chronic or recurrent episodes [1, 3]. Among the factors described in the literature is immunosuppression, which constitutes the main feature of patients undergoing hematopoietic stem cell transplantation. The immunosuppression of these patients is therefore believed to be the only cause for their high frequency of rhinosinusitis [1, 3–6].

The frequency of bacterial rhinosinusitis in patients receiving hematopoietic stem cell transplants (HSCTs) is much higher (37%) than that of immunocompetent patients (5 to 15%) [2, 6–10]. The risk is further increased in HSCT patients who develop chronic graft versus host disease (GVHD), with a 4.3 times higher frequency of infection. In addition, there is a risk of developing the dreaded invasive fungal rhinosinusitis, although the frequency of this type is lower (0.3 to 3.8%) [11–15].

Previous studies have shown histological and ultrastructural abnormalities in the nasal mucosa of patients with chronic GVHD undergoing HSCT compared with immunocompetent patients, as described in Table 1. This epithelial damage could be another causal feature for the high prevalence of sinus infections in transplanted patients [16, 17].

**Table 1:** **Histology of the nasal mucosa**

	HSCT	HSCT with GVHD	Immunocompetent
Cilia (absence or decrease) (%)	77	76	0
Ciliar ultrastructure abnormalities (%)	50	18	33
Cytoplasmic mitochondria (decreased/absence) (%)	80	47	0
Cytoplasmic vacuolization (%)	60	35	0
Apoptotic bodies	2.3	6.6	0.75
Goblet cells (decreased/absence), *n* (%)	2 (40%)	9 (53%)	2 (22%)
Moderate/severe inflammatory infiltrate, *n* (%)	13 (100%)	8 (62%)	7 (70%)

Comparisons among patients undergoing hematopoietic stem cell transplant (HSCT), with and without graft versus host disease (GVHD), and immunocompetent patients.

A subsequent study showed that there was no change in the frequency of nasal epithelial damage in the presence of rhinosinusitis in patients undergoing HSCT, even among those with GVHD. Immunosuppression thus remains the main cause of the higher frequency of rhinosinusitis in HSCT and GVHD [18].

Nevertheless, it seems that the recurrence of nasal infection causes a progressive decrease in the number of cilia in the sinus epithelia of transplanted patients [18]. Therefore, it is possible that epithelial damage could be an associated or aggravated feature of sinusitis recurrence or chronicity.

### Objective

Because transplanted patients have histological abnormalities after the hematopoietic stem cell transplantation process, as well as in the presence of rhinosinusitis, and because the recurrence of rhinosinusitis increases this epithelial damage, the aim of this study is to verify the influence of rhinosinusitis on nasal epithelial damage in patients undergoing hematopoietic stem cell transplantation.

## Methods

This exploratory prospective study includes patients who underwent a transplant of hematopoietic stem cells at the Transplantation Hematopoietic Stem Cells Unit of the HC UNICAMP/Blood Center of Campinas and who were evaluated by the Office of Rhinology of Otorhinolaryngology, FCM-UNICAMP, University of Campinas, Brazil. The study was approved by Research Ethics UNICAMP, under number 088-2002, and patients also completed the research ethics consent form to participate in this study.

We selected a total of 30 allogeneic hematopoietic stem cell-transplanted patients who were divided into two groups: 24 patients with chronic or recurrent rhinosinusitis, and six patients without rhinosinusitis. These patients underwent a biopsy of the nose to collect the samples; 24 patients underwent biopsies in the presence of rhinosinusitis. Only 22 slides for the group with rhinosinusitis were analyzable by electron microscopy, and 19 slides were examined by optical microscopy (24 patients). The biopsies of the six patients without rhinosinusitis were analyzed by electron microscopy, and five were examined by optical microscopy.

The rhinosinusitis diagnosis was made by clinical and endoscopic nasal evaluation, according to the criteria of the Brazilian Guidelines on Rhinosinusitis and a European Position Paper [1, 2].

Exclusion criteria included patients with serious risks of bleeding due to thrombocytopenia and patients with cytomegalovirus or herpes virus.

Thirty patients, with an average age of 39 years and a male predominance (63%), were transplanted with allogeneic hematopoietic stem cells. In total, 73% of these patients underwent myeloablative transplantation and 27% underwent nonmyeloablative transplantation. Table 2 presents the general characteristics of the groups of transplanted patients, with and without recurrent or chronic rhinosinusitis.

**Table 2:** **Groups of transplanted hematopoietic stem cells**

	HSCT	Hematologic disease	GVHD
With rhinosinusitis	Myeloablative: 16 (66%)	CML: 5 (21%)	With: 16 (66%)
		AML: 5 (21%)	
	Nonmyeloablative: 8 (33%)	MM: 3 (12.5%)	Without: 8 (33%)
		CLL: 1 (4%)	
		AA: 3 (12.5%)	
		MDS: 4 (17%)	
		ALL: 2 (8%)	
		SCA: 1 (4%)	
Without rhinosinusitis	Myeloablative: 6 (100%)	ALL: 1 (17%)	With: 5 (83%)
		CML: 3 (50%)	
		AML: 1 (17%)	Without: 1 (17%)
		AA: 1 (17%)	

AA, aplastic anemia; ALL, acute lymphoblastic leukemia; AML, acute myeloid leukemia; CLL, chronic lymphocytic leukemia; CML, chronic myeloid leukemia; GVHD, graft versus host disease; HSCT, hematopoietic stem cell transplant; MDS, myelodysplastic syndrome; MM, multiple myeloma; SCA, sickle cell anemia.

Biopsies of the uncinate process mucosa at the middle meatus were endoscopically performed for all patients under anesthesia [15]. Those biopsies were taken from the full thickness of the uncinate process of those patients that underwent to surgery; and two or three samples from the medial to lateral uncinate process, with cutting blaksley. Histopathological evaluations were achieved using optical microscopy and transmission electron microscopy. Hematoxylin and eosin, Periodic acid–Schiff and Masson’s trichrome staining were used to better assess each variable. The histological criteria analyzed by light microscopy of hematoxylin and eosin-stained samples were the density and composition of the epithelial and submucosal inflammatory infiltrate, the number of apoptotic bodies and intraepithelial eosinophils, and the submucosal glandular density. The intraepithelial lymphocyte counts and the density of the epithelial and submucosal inflammatory infiltrate were examined by Periodic acid–Schiff, and the basal membrane thickening, submucosal edema and fibrosis were determined by Masson’s trichrome stain. The variable counts were determined by analyzing the histological sections that were randomly selected from the best embedded glass slides using at least 10 fields per 40× high-power field. The measurements of the basal membrane were made with the aid of a micrometer eyepiece graticule (Breslow strip) in histological sections stained with Masson’s trichrome in 10 high-power fields of 40× magnification, which were randomly selected areas where the material was well embedded. The lymphocyte, eosinophil, apoptotic body and submucosal gland counts, as well as the basal membrane thickness values, were summed and divided by the number of selected fields (arithmetic average) for a comparative analysis among the groups. The inflammatory infiltrate density as well as edema and fibrosis levels were subjectively graded under optical microscopy as absent, mild, moderate or severe.

For electron microscopy, the sample was placed on a permeable thin paper card on a hard wax and cut into fine fragments approximately 0.3 mm in diameter and then placed in a container with fixative (3% glutaraldehyde solution) and maintained at 4°C for 3 hours. The samples were then processed through the wash steps and placed in a container with phosphate buffer. Various fragments of approximately 2 mm from the uncinate process were randomly selected and made into ultrathin sections 50 to 60 nm thick using an ultramicrotome diamond knife. The magnification used was 1,600 to 20,000×. The parameters subjectively evaluated by transmission electron microscopy included the following: amount of cilia (absent, decreased, normal), organization of ciliary structures, squamous metaplasia (absent or present), microvilli (present or absent), goblet cells (absent, decreased, increased or normal), intracellular mitochondria (present or absent) and cytoplasmic vacuolization (present or absent). The ciliary orientation was measured by cross-sections of at least 10 cilia, which were delineated as one perpendicular line between the central microtubules and arranged in the same direction and angle within a possible variation of 10°.

For light microscopy, all of the slides were examined by two pathologists, and for electron microscopy, all of the fragments of the biopsy were always evaluated by the same investigator.

The data collected from the biopsies were compared among the two groups using a statistical analysis of contingency (chi-squared) and the Wilcoxon test in R version 2.7.0 (© 2008 The R Foundation for Statistical Computing). The level of significance (*P* value) was set at 0.05, and confidence intervals were set at 95%.

The data used as a control for comparison and discussion in this study were obtained from our previous studies, the methodologies of which were identical [11]. These studies evaluated the histological aspects of the nasal mucosa of immunocompetent patients and patients undergoing bone marrow transplants, with and without rhinosinusitis [16, 18].

## Results

**Figure 1:**
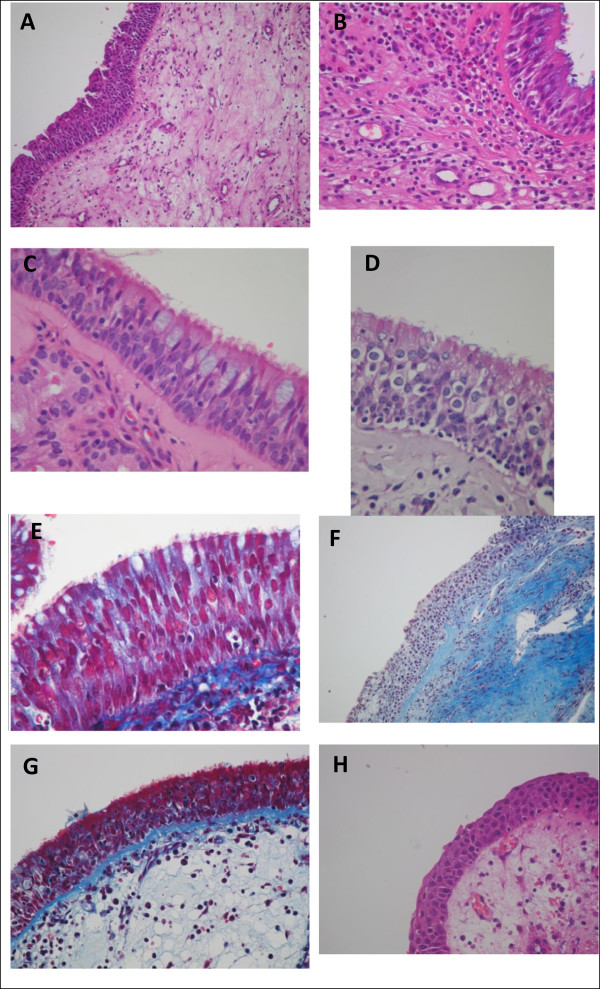
**Histological sections of the nasal epithelia of transplanted patients under optical microscopy. (A)**, **(B)** Intense and moderate eosinophilic inflammatory infiltrate and submucosal epithelium (hematoxylin and eosin). **(C)**, **(D)** Lymphocytic inflammatory infiltrate epithelium (Periodic acid–Schiff). **(E)** Ciliated epithelium, pseudostratified without fibrosis (Masson’s trichrome). **(F)** Subepithelial (Masson’s trichrome) fibrosis. **(G)** Thickening of the basement membrane (arrow) in Masson’s trichrome. **(H)** Submucosal edema (Hematoxilin and eosin).

There were no significant changes between the groups of HSCT patients, with and without rhinosinusitis, in any of the variables analyzed in the nasal mucosal analysis by optical microscopy (Table 3). However, the density of the inflammatory infiltrate in patients with rhinosinusitis was moderate in 37% of the patients, compared with no evidence of inflammatory infiltrate in transplanted patients without rhinosinusitis. The mucosal eosinophil and lymphocytes counts were almost 10 times higher in the HSCT patients with rhinosinusitis. Severe edema and fibrosis occurred in 16% of the patients with rhinosinusitis and occurred in none of those without rhinosinusitis. Figure 1 shows the histological sections under optical microscopy, comparing the HSCT patients with and without rhinosinusitis.

**Table 3:** **Analysis of the sinonasal epithelium by optical microscopy**

Light microscopy	Without RS	With RS	***P*** value
Moderate inflammatory infiltrate	0	7 (37%)	0.73
Intraepithelial lymphocytes (*n*/HPF)	1.14	12	0.073
Apoptotic bodies (*n*/HPF)	2.3	6.6	0.498
Eosinophils (*n*/HPF)	1.4	14.4	0.155
Glandular density (*n*/HPF)	8.6	11	0.227
Moderate/severe edema	0	3 (16%)	1
Fibrosis	0	31 (16%)	0.822
Basal membrane (mm)	0.019	0.048	0.347

Patients with transplanted hematopoietic stem cells, with and without rhinosinusitis (RS). Fisher’s exact test and Wilcoxon analysis with *P* <0.05. *n*/HPF = number per high-power field of greatest increase.

**Figure 2:**
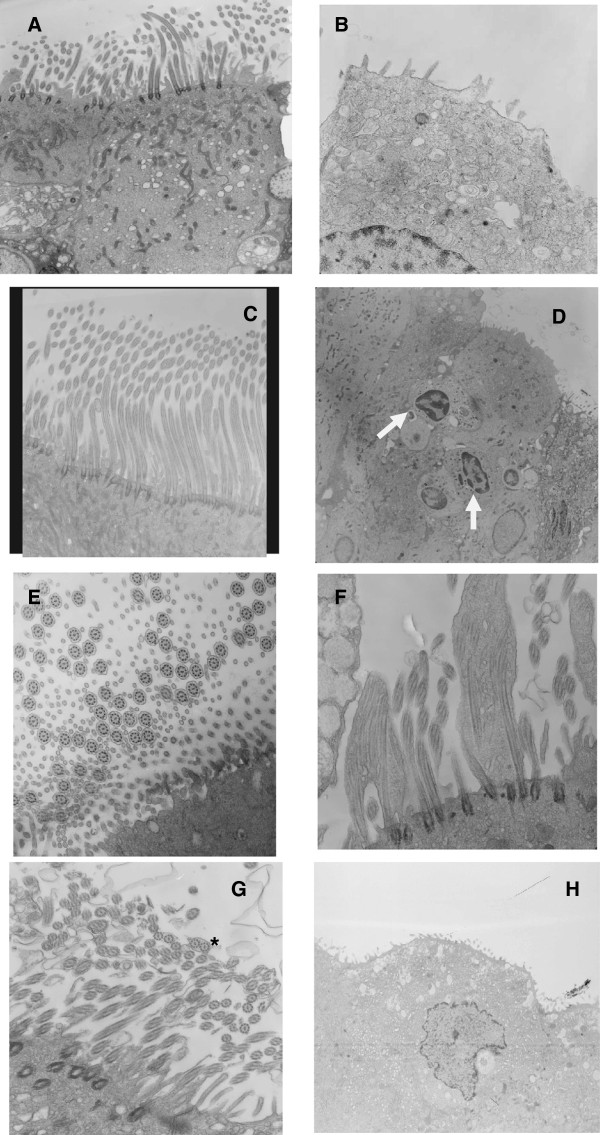
**Ciliated nasal epithelium of transplanted patients without and with rhinosinusitis under transmission electron microscopy. (A)** Ciliated cells with a cilia decrease and microvilli increase. **(B)** Ciliated cells without cilia or mitochondria and with cytoplasmic vacuolization. **(C)** Normal ciliated epithelium with goblet cells. **(D)** Ciliated epithelium without cilia and mononuclear cells migrating to the surface (arrows). **(E)** Epithelium with normal cilia. **(F)** Compound cilia (longitudinal). **(G)** Compound cilia (*transversal). **(H)** Squamous metaplasia. Right column, patients without rhinosinusitis; left column, patients with rhinosinusitis.

Regarding the parameters studied by transmission electron microscopy, there was a significant difference in the normal ciliary orientation in the group without rhinosinusitis. There was a trend toward a difference in the amount of cilia (a decreasing trend) and the primary modification of the ultrastructure in those with rhinosinusitis (Table 4). The primary modifications of the ultrastructure were the compound cilia and triple central microtubules. Forty-five percent of the patients with rhinosinusitis had no cilia on the surfaces of the ciliated cells, whereas the cilia were not absent in the HSCT patients without rhinosinusitis. Squamous metaplasia occurred in approximately 23% of the patients in the rhinosinusitis group. Despite there being no significant difference in the number of goblet cells, approximately 59% of transplanted patients with rhinosinusitis had few or no goblet cells in the nasal respiratory epithelium. Further, 55% of transplanted patients with rhinosinusitis had decreased amounts of cytoplasmic mitochondria. Figure 2 shows the histological sections of the different groups under electron microscopy.

**Table 4:** **Analysis of the nasal epithelium by transmission electron microscopy**

Electron microscopy	Without RS	With RS	***P*** value
Cilia (absence)	**0**	**13 (59%)**	**0.052**
Ultrastructural modification	**2 (33%)**	**5 (50%)**	**0.054**
Normal ciliary orientation	**5 (83%)**	**8 (36%)**	**0.009**
Squamous metaplasia	0	5 (23%)	0.553
Microvilli	5 (83%)	16 (73%)	1
Goblet cells (decreased/absence)	1 (17%)	13 (59%)	0.306
Cytoplasmic mitochondria (decreased/absence)	1 (17%)	12 (55%)	0.285
Cytoplasmic vacuolization	3 (50%)	10 (45%)	1

Hematopoietic stem cell transplanted patients, with and without rhinosinusitis (RS). Fisher’s exact test and Wilcoxon analysis with *P* <0.05 are in bold.

## Discussion

Compared with immunocompetent patients, there is a higher prevalence of bacterial rhinosinusitis in HSCT patients; recurrence is 4.3 times higher in HSCT patients with chronic GVHD [6–8].

A previous study found that hematopoietic stem cell-transplanted patients experience epithelial injury after transplantation, showing a reduction in the number of cilia (77%) and goblet cells (50%) and a 50% change in the ciliary ultrastructure, independent of sinonasal infections [16]. These abnormalities would trigger mucous dryness, malfunctions of mucociliary clearance, and inadequate lubrication that prevents the surface maintenance of defense proteins (lysozymes, lactoferrins and IgA) [19]. This epithelial damage, in addition to the immunosuppression inherent in transplantations, may explain the higher rates of occurrence and recurrence of rhinosinusitis in these patients.

Chronic GVHD can affect the mucosal membranes of transplanted hematopoietic stem cell patients due to a cytotoxic reaction that results in the generation of apoptotic bodies as the end product of cellular degeneration in the epithelium [20]. Previous studies have shown an increase in the number of apoptotic bodies in the sinonasal mucosa of HSCT with chronic GVHD [16]. This result could also explain the greater risk of rhinosinusitis in these patients, as described in the literature.

However, a subsequent study showed no difference in the epithelial damage in HSCT patients, with or without chronic GVHD, in the presence of rhinosinusitis [18]. The only significant finding of this study was an increase in the number of microvilli, which may have occurred due to release of proinflammatory cytokines in the nasosinal mucosa in chronic rhinosinusitis. These cytokines decrease ciliogenesis and the formation of basal bodies, preventing the elongation of the centrioles [21]. A transplanted patient with chronic GVHD does not seem to have more severe epithelial lesions relative to other HSCT patients, although such a patient would appear to have more edema and fibrosis. In addition, these patients appear to have denser inflammatory infiltrate with eosinophils and decreased glandular density in the submucosa. It has been suggested that immunosuppression remains the main cause of the higher prevalence of rhinosinusitis among recipients, with or without GVHD [18].

In immunocompetent patients, chronic and/or recurrent rhinosinusitis is known to cause histological abnormalities in the sinus mucosa, disrupting the protective barrier and potentially triggering the immune response of the individual, depending on the pathogen [22]. These abnormalities may be ultrastructural (17 to 45%), related to the leukocyte inflammatory infiltrate, the epithelial squamous metaplasia, the glandular hyperplasia or the presence of microvilli [22–25]. Aside from these abnormalities, aggravating factors – such as anatomical variations, allergy, smoking and genetic diseases – can perpetuate sinonasal inflammation or stimulate its recurrence [1, 2]. A previous study showed that rhinosinusitis recurrence in HSCT patients is associated with an absence or decrease in the number of cilia [18]. Rhinosinusitis may thus be an aggravating factor for sinonasal epithelial damage in these patients.

In this study, rhinosinusitis did not significantly influence the nasal histology of HSCT patients, although these patients did show a more dense inflammatory infiltrate with eosinophils and intraepithelial lymphocytes, as well as increased edema and fibrosis. These features seem to result from the modification of the top of the cilia and the ciliary ultrastructure (50%), which changes the orientation of the cilia during rhinosinusitis.

The ultrastructural abnormalities found here are the same as those in chronic rhinosinusitis among immunocompetent patients, including compound cilia and triple central microtubules, which occur after the release of cytokines or toxins and proteases from the infecting microorganisms (viruses, bacteria or fungi). These abnormalities may improve over an approximately 10-week course of recovery from infection [10, 21, 26].

Epithelial damage may therefore be a consequence of the transplant process and an aggravating factor for rhinosinusitis. Rhinosinusitis recurrences can also act as an aggravating factor for this epithelial damage. Finally, immunosuppression is the main causal factor for HSCT rhinosinusitis and chronic GVHD.

## Conclusion

HSCT patients, with and without rhinosinusitis, showed no significant histological abnormalities, except for ciliary disorientation, a possible decrease in the amount of cilia and an increase in ultrastructural abnormalities in HSCT patients with rhinosinusitis.

## Abbreviations

GVHD: graft versus host disease
HSCT: hematopoietic stem cell transplant.
